# Mind-Body Practice, Personality Traits, and Cognitive Performance: A 10-Year Study in US Adults

**DOI:** 10.1093/geroni/igab046.2651

**Published:** 2021-12-17

**Authors:** Kallol Kumar Bhattacharyya, Gizem Hueluer, Debra Dobbs

**Affiliations:** 1 School of Aging Studies, University of South Florida, Tampa, Florida, United States; 2 University of Bonn (Germany); 3 University of South Florida, School of Aging Studies, University of South Florida, Florida, United States

## Abstract

It is widely established that physical activity is associated with better cognitive outcomes, and accumulating evidence suggests that mind-body practice (MBP) may yield similar benefits. Personality is related to both daily activities and cognition, but its role in the association between MBP and cognition is not well understood. The current study examines bidirectional temporal associations between personality traits, MBP, and cognition in healthy adults. We used data from waves 2 and 3 (2004-2014) of the Midlife in the United States (MIDUS) study from a total of 2,050 individuals (age: M=64 years, SD=11, range=42 to 92; 56% women). We applied a cross-lagged regression analysis to examine bidirectional effects between MBP, Big Five personality traits, and two cognitive domains (episodic memory and executive function) and controlled for sociodemographic factors, health, and functional status covariates in wave 2. After controlling for covariates, MBP was independently associated with a more favorable change in episodic memory, but not in executive function. Regarding cross-lagged effects of cognitive function, episodic memory was related to subsequent MBP and agreeableness, and executive function was related to subsequent MBP, openness, and conscientiousness. Agreeableness had a negative effect on subsequent executive function. The findings point toward bidirectional associations between cognitive function MBP, while there was no evidence for cross-lagged associations between personality and MBP. Future research should guide us whether MBP can counteract cognitive decline as an alternative and complementary practice and the role that personality can play in such interventions.

